# Efficacy of a hypolipid diet in patients with primary antiphospholipid syndrome with dyslipidemia: a prospective study

**DOI:** 10.1007/s11239-021-02542-z

**Published:** 2021-08-21

**Authors:** Thays C. R. Rodrigues, Camila de Oliveira Vaz, Eliana C. M. Miranda, Marcos Pereira, Sabrina da Silva Saraiva, Joyce Maria Annichino-Bizzacchi, Bruna de Moraes Mazetto, Fernanda A. Orsi

**Affiliations:** 1 Hematology and Hemotherapy Center, University of Campinas, Campinas, SP Brazil; 2 Department of Clinical Pathology, School of Medical Sciences, University of Campinas, Campinas R. Tessália Vieira de Camargo, 126. Cidade Universitária, Campinas, SP 13083-887 Brazil; 3 Leiden University Medical Center (LUMC), Leiden, The Netherlands

**Keywords:** Dyslipidemia, Antiphospholipid syndrome, Diet therapy, Atherosclerosis, Cardiovascular risk, Hypolipid diet

## Abstract

Although dyslipidemia is associated with poorer prognosis in antiphospholipid syndrome (APS), the management of lipid disorders can be challenging. While statins may increase the bleeding risk associated with anticoagulation, the effectiveness of hypolipid diet (HD) has not yet been established in patients with autoimmune disorders. In this study, we evaluated whether HD is associated with decreases in cholesterol levels in patients with thrombotic primary APS (t-PAPS) and dyslipidemia. Nutritional and lipid profiles were assessed before HD was initiated (baseline) and after 3 and 6 months with HD. A 24-h dietary recall was applied to assess the adherence to the diet. Forty-four patients were included, mean age was 43 years (± 12.93) and 65% were female. After HD was started, the intake of carbohydrates, lipids, saturated fats and cholesterol decreased, whereas dietary fiber intake increased. Levels of total cholesterol (TC) and non-high density lipoprotein cholesterol (non-HDL-C) decreased after 3 and 6 months of HD, as compared to baseline (P = 0.007 and P = 0.008). Low-density lipoprotein cholesterol (LDL-C), triglycerides (TG), and high-density lipoprotein cholesterol (HDL-C) values did not change during the study period. The mean body mass index (BMI) decreased from 28.4 to 27.8 kg/m^2^ after six months of HD (p < 0.0001). In subgroup analysis, the effects of HD were more pronounced in patients with high TC, LDL-C or non-HDL-C levels at baseline and in those without obesity or hypertension. Nutritional intervention is feasible among t-PAPS and could be an alternative therapy to modulate lipid metabolism in this population.

## Highlights

- The management of lipid disorders in primary antiphospholipid syndrome (aps) is challenging.
- Statins may increase the bleeding risk associated with anticoagulation and the effectiveness of hypolipid diet is not established.
- In a cohort of primary APS, levels of cholesterol and body mass index decreased with hypolipid diet.
- Nutritional intervention is feasible and effective for treatment of lipid disorders in primary APS.

## Background

Antiphospholipid syndrome (APS) is an autoimmune disorder characterized by thromboembolic events or obstetric complications, such as fetal loss, recurrent miscarriages or preeclampsia. APS occurs either as a primary disorder (PAPS) or secondary to other autoimmune diseases, usually systemic lupus erythematosus (SLE) [1, 2]. Thrombotic APS is the vascular form of the disease, which is characterized by venous, arterial or capillary thrombosis associated with persistently positive antiphospholipid antibodies (aPL). These are antibodies directed against proteins that bind to cell membrane phospholipids, are responsible for the pathological mechanisms in APS [3], as they induce procoagulant and proinflammatory states [4]. Apart from the presence of aPL, additional prothrombotic mechanisms are required to trigger thrombosis, such as hypertension, smoking, obesity, diabetes, physical inactivity and dyslipidemia [5]. Hypertension and dyslipidemia have been associated with the risk of thrombosis in patients with APS. Hypertension and dyslipidemia have been associated with the risk of thrombosis in patients with APS. The GAPSS score, which is a clinically validated tool to predict the risk of thrombosis in APS patients, includes both hypertension and dyslipidemia, along with the presence of aPL, as independent determinants of thrombosis risk in APS [6].

Dyslipidemia is a condition caused by dysregulation of the lipid metabolism and is associated with an increased risk of atherosclerosis and cardiovascular events [7, 8], such as coronary artery disease and stroke [9]. In patients with APS, dyslipidemia may further aggravate the underlying risk of thrombosis conferred by the presence of aPL [10]. Statin therapy is the treatment of choice to control lipid levels and reduce cardiovascular morbidity. However, statin use may be challenging in APS patients with thrombosis since statins interact with vitamin K antagonists, the main anticoagulant drug used by these patients [4, 11], increasing the risk of bleeding [12].

In this context, a hypolipid diet (HD) emerges as a potential therapy for the treatment of dyslipidemia [13, 14], particularly in cases when statin use is contraindicated. Nutritional intervention through a balanced diet can be used for the control of chronic non communicable diseases, such as dyslipidemia, and several studies have shown that HD is effective in reducing the risk of cardiovascular diseases [13, 14]. However, HD may be less effective at controlling lipid levels in cases in which dyslipidemia is associated with an autoimmune disease, such as SLE [15, 16]. The efficacy of HD in PAPS patients with dyslipidemia has not yet been evaluated.

Therefore, the aim of this study was to evaluate whether HD is associated with a decrease in the levels of total cholesterol (TC) and non-high density lipoprotein cholesterol (non-HDL-C) in a group of patients with thrombotic primary APS (t-PAPS) and dyslipidemia. We further evaluated whether the diet is associated with a reduction in the levels of low-density lipoprotein cholesterol (LDL-C), high-density lipoprotein cholesterol (HDL-C), and triglycerides (TG).

## Methods

### Study design and participants

This is a single-arm, prospective, open, single-center cohort study. The cohort consisted of consecutive patients with thrombotic PAPS (t-PAPS) and dyslipidemia treated at the outpatient clinic of the Hematology and Hemotherapy Center of the University of Campinas. The selection of subjects was carried out after approval of this study by the Ethics and Research Committee of this University and National Registry of Clinical Studies—U1111-1242-4151, with the Free and Informed Consent Form (ICF) being presented and only after acceptance was it given beginning of this research, from August 1, 2016 to June 30, 2017. Inclusion criteria included: diagnosis of t-PAPS and dyslipidemia. Patients diagnosed with systemic autoimmune disease (other than APS) or neoplasia or aged below 18 years were excluded from the study.

Dyslipidemia was characterized as LDL-C levels above 100 mg/dL, HDL-C levels below 40 mg/dL (men) or 50 mg/dL (women), TG levels above 200 mg/dL, non-HDL-C levels above 130 mg/dL and TC levels above 190 mg/dL. The cut-off levels to determine dyslipidemia were chosen in face of the fact that the patients had a previous history of venous thrombosis or stroke. The threshold of the lipid levels that indicate treatment in patients with previous cardiovascular disease is lower than that in patients without these conditions, as recommended by both the American and the Brazilian Societies of Cardiology [17, 18].

At the day of inclusion, patients were submitted to a nutritional assessment and lipid profile tests (TC, LDL-C, HDL-C, non-HDL, TG). Demographic and clinical characteristics were assessed by interviewing patients and reviewing medical records. The nutritional status of patients was assessed by measuring waist circumference, triceps skinfold thickness, and body mass index (BMI). The nutritional status was classified as underweight, normal weight, overweight, and obesity according to World Health Organization criteria [19].

### Nutritional intervention

During nutritional appointments in the Centre of Hematology, each patient was instructed to follow an individualized diet in which the total caloric value (VCT) was calculated according to the need for weight maintenance or loss. The diet was delivered in a printed guide, containing a schedule of meals and preparations. The amount of calories in the diet was calculated considering the total energy expenditure in calories per kilogram of weight per day (20 to 25 kcal/kg per day) [20]. Five to six meals per day were recommended (breakfast, snack, lunch, snack, dinner, and supper) and controlled food volume was encouraged. In addition, the consumption of two to four portions of fruit and two portions of raw or cooked vegetables was recommended.

There was no group division, each patient was treated as a single case, the caloric distribution of nutrients was determined according to the dietary recommendations for the treatment of dyslipidemia proposed by the American Heart Association [21], the I Guideline on Fat Consumption and Cardiovascular Health [22] and the IV Brazilian Guideline for Dyslipidemia and Prevention of Atherosclerosis [23]. Briefly, the daily recommended intakes are: total fat 25–35%; saturated fatty acids ≤ 7%; polyunsaturated fatty acids ≤ 10%; monounsaturated fatty acids ≤ 20%; carbohydrates 50 to 60%; proteins 15%; cholesterol < 200 mg; and fiber 20 to 30 g. As there is no consensus regarding the maximum amount of trans-fat consumption, the recommendation followed less than 1% of total dietary calories.

After introducing HD, patients were reevaluated at 3 and 6 months with nutritional consultations and laboratory tests. Clinical data collected during consultations were: anthropometric assessment and food history to provide reorientation when needed. The Research Ethics Committee of the School of Medical Sciences of the University of Campinas approved this study (CAAE: 53655916.7.0000.5404) and study registered in the national registry of clinical trials under number U1111-1242-4151, after the presentation and signature of the Informed Consent Form, the research started (2016/08—2017/06).

### Statistical analysis

Baseline characteristics are presented as numbers and percentages in the case of categorical variables or mean ± standard deviation (SD) in the case of continuous variables. Mean values (and SD) of all studied parameters (TC, LDL-C, HDL-C, non-HDL, TG and BMI) were calculated before HD was introduced (baseline) and at 3 months and 6 months of HD. We compared the levels of LDL-C, HDL-C, non-HDL, TG and BMI between the three time-points using ANOVA test. We then divided the patients in subgroups according to baseline lipid levels and diagnosis of obesity or hypertension and applied the same strategy to evaluate changes in the levels of TC, LDL-C, HDL-C, non-HDL, TG and BMI during HD in these subgroups.

To evaluate the association of HD and dichotomous outcomes (nutritional status classification, risk of metabolic complications, physical activity, and statin use), we calculated the frequency (in percentage) of these parameters at baseline and at 6 months of HD. These frequencies were compared using the chi-squared test.

All statistical analyses were performed with SPSS version 23.0 for Windows (SPSS Inc, IBM, Armonk, NY, USA).

## Results

**Fig. 1:**
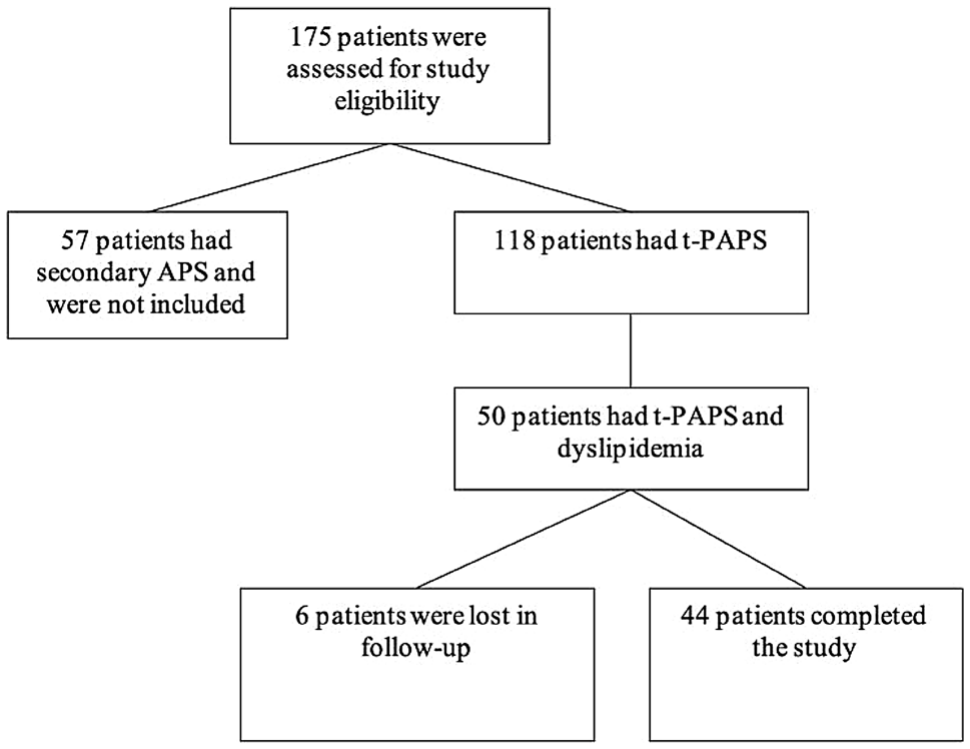
Flowchart of the study. *t-PAPS* patients with thrombotic primary antiphospholipid syndrome

Fifty t-PAPS patients were enrolled for the study. Figure 1 illustrates the flowchart of the study. Clinical characteristics of the patients are described in Table 1. The mean age was 43 years-old (SD 12.93), 29 patients (65.9%) were female, 17 (38.6%) had hypertension, 7 (15.9%) reported smoking or regular alcohol intake, 10 patients (22.7%) were using statins and 13 (29.5%) acetylsalicylic acid (ASA). No other therapy to reduce lipid levels, such as fibrate, ezetimibe, PCSK9 inhibitors, was given to the patients. With regards to aPL profile, 16 patients (36.36%) were positive for just one aPL, 15 (34.09%) patients were double positive and 13 (29.55%) were triple aPL positive. All patients had a history of prior thrombosis, mainly venous thrombosis (n = 33; 75%).

**Table 1:** Demographic and clinical characteristics of patients

Characteristics		
Sex, n (%)		
Male	15	(34.1)
Female	29	(65.9)
Comorbidities, n (%)		
Hypertension	17	(38.6)
Diabetes	02	(4.5)
Metabolic syndrome	27	(61.4)
Dyslipidemia	44	(100.0)
Statins use, n (%)	10	(22.7)
Aspirin use, n (%)	13	(29)
Type of thrombosis, n (%)		
Venous Thrombosis	33	(75.0)
Stroke	08	(18.2)
Arterial and Venous thrombosis	02	(4.5)
Peripheral Arterial disease	01	(2.3)
Smoking or alcohol intake, n (%)	07	(15.9)
TSH^a^, median (IQR)	2.32	(1.56–3.81)

*n* absolute number of patients, *TSH* thyroid *stimulating hormone*

^a^TSH results were available for 30/44 patients

The levels of TC, non-HDL-C, LDL-C, TG and HDL-C at baseline and at 3 and 6 months of HD are described in Table 2. Mean TC levels decreased from 192.16 mg/dL (SD 35.23) at baseline to 183.33 mg/dL (SD 42.32) at 3 months and 179.78 mg/dL (SD 43.46) at 6 months of HD (P = 0.007), resulting in a 7% decrease in TC levels from baseline until the end of study. Mean non-HDL-C levels decreased from 144.41 mg/dL (SD 33.19) at baseline to 136.38 mg/dL (SD 38.21) at 3 months and 133.07 mg/dL (SD 40.95) at 6 months of HD (P = 0.02), resulting in 7.85% decrease in non-HDL-C levels from baseline until the end of study. There was no change in LDL-C, TG and HDL-C levels after the HD nutritional intervention was started. Values of BMI also decreased during HD therapy. Mean BMI values was 28.41 kg/m^2^ (SD 5.58) at baseline, 27.89 kg/m^2^ (SD 5.40) after 3 months of HD and 27.81 kg/m^2^ (SD 5.55) after six months of HD (P < 0.0001).

**Table 2:** Lipid levels at baseline and at 3 and 6 months with HD

Lipids	Baseline		3 months		6 months		P*
	Mean	SD	Mean	SD	Mean	SD	
TC	192.16	(35.23)	183.33	(42.32)	179.78	(43.46)	0.007
Non-HDL-C	144.41	(33.19)	136.38	(38.21)	133.07	(40.95)	0.02
LDL-C	115.38	(30.58)	109.15	(34.41)	106.75	(34.82)	0.16
TG	143.81	(83.99)	136.06	(82.66)	131.52	(88.57)	0.10
HDL-C	47.75	(14.14)	46.95	(12.11)	46.70	(10.79)	0.24

*HDL-C* high-density lipoprotein cholesterol, *LDL-C* low-density lipoprotein cholesterol, *Non-HDL-C* non-HDL cholesterol, *SD* standard deviation, *TC* total cholesterol, *TG* triglycerides;

^*^P values were calculated using ANOVA test

Nutritional intervention with HD resulted in decreased intake of dietary lipids, saturated fat, dietary cholesterol and total calories. From baseline until the end of the study, the intake of lipids decreased from 64.85 (SD 13.30) to 57.64 g (SD 14.28; P = 0.002), saturated fat intake decreased from 22.89 (SD 6.85) to 19.40 g (SD 6.28; P = 0.002), dietary cholesterol decreased from 298.03 (SD 145.87) to 232.40 mg (SD 84.68; P = 0.007) and total calories decreased from 2159.18 (SD 388.82) to 2092.65 kcal (SD 402.59; P = 0.032). Dietary fiber intake increased from 39.68 g (SD 18.26) at baseline to 45.38 g (SD 17.38; P = 0.005) after 6 months with HD. There was no change in the amount of carbohydrate (P = 0.06), protein (P = 0.59), monounsaturated fat (P = 0.07), polyunsaturated fat (P = 0.48), and sugar (P = 0.64) intake with HD.

### Subgroup analysis

We analyzed the effect of HD in subgroups of patients with baseline levels of TC below and above 190 mg/dL, non- HDL-C below and above 130 mg/dL, LDL-C below and above 100 mg/dL and TG below and above 200 mg/dL.

**Fig. 2:**
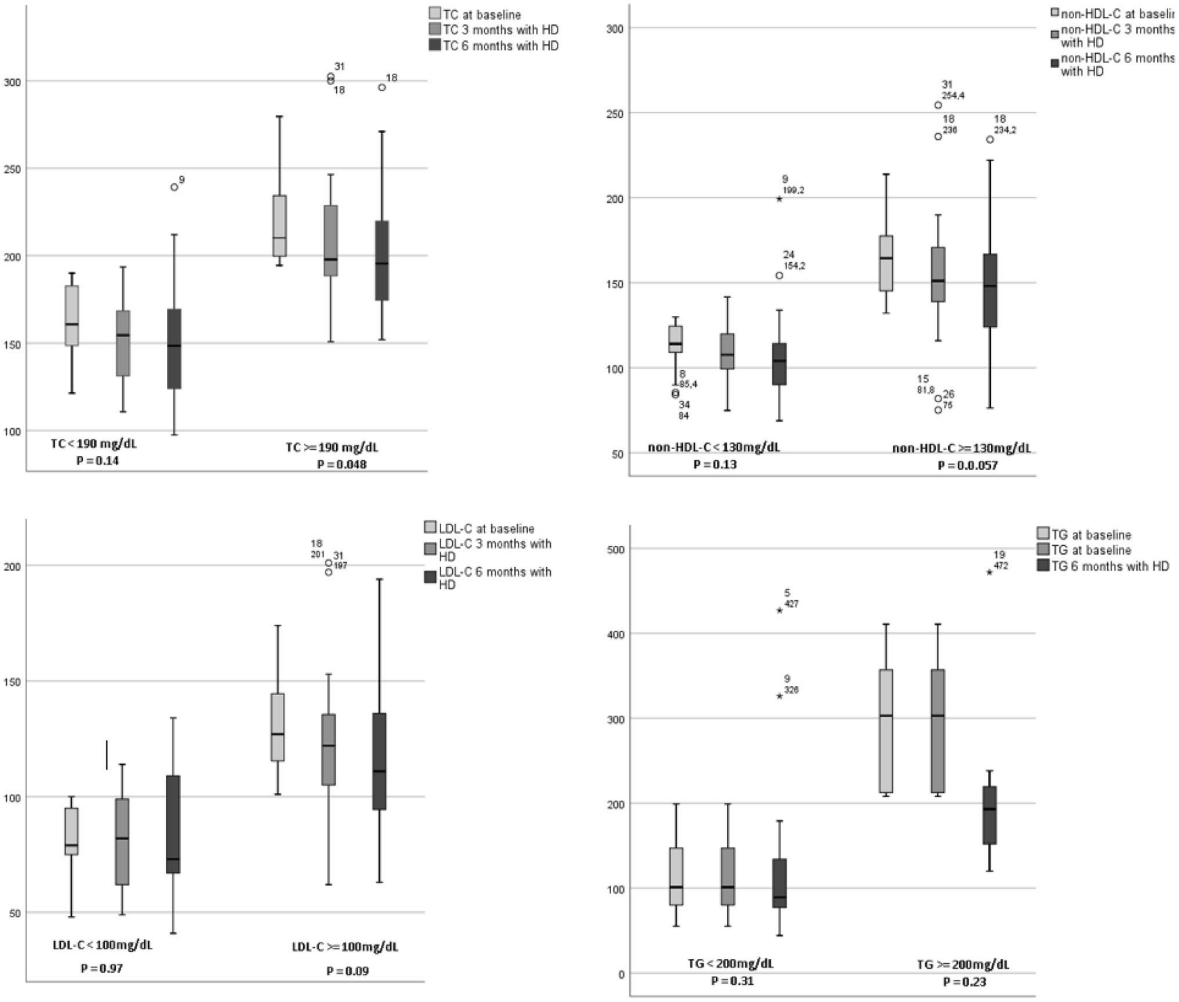
Changes in the levels of TC, LDL-C, HDL-C, non-HDL, TG in subgroups of patients divided according to the baseline levels of lipids. *SD* standard deviation, *TC* total cholesterol, *Non-HDL-C* non-HDL cholesterol, *LDL-C* low-density lipoprotein cholesterol, *TG* triglycerides, *HDL-C* high-density lipoprotein cholesterol. P values were calculated using ANOVA test

Figure 2 illustrates the results. In patients with TC levels ≥ 190 mg/dL at baseline, TC levels decreased from baseline to 6 months with HD therapy (P = 0.048), whereas in patients with baseline TC levels < 190 mg/dL, TC levels did not change substantially during HD (P = 0.14). Non-HDL-C levels decreased from baseline to 6 months with HD only in patients with baseline non-HDL-C levels ≥ 130 mg/dL (P = 0.057), in patients with baseline non-HDL-C levels < 130 mg/dL no significant change in non-HDL-C levels was observed during HD therapy (P = 0.13). LDL-C levels decreased with HD in patients with baseline LDL-C levels ≥ 100 mg/dL (P = 0.09) though not in patients with baseline LDL-C levels < 100 mg/dL (P = 0.97). Levels of TG decreased only in patients with baseline TG ≥ 100 mg/dL, the difference however was not statistically significant.

In overweight/obese patients (baseline BMI > 24.9 kg/m^2^), mean BMI values decreased from 31.59 kg/m^2^ (SD 3.96) at baseline to 31.03 kg/m (SD 3.66) at 3 months and 30.95 kg/m^2^ (SD 3.99) at 6 months of HD (P = 0.002). In the subgroup of eutrophic patients (BMI < 24.9 kg/m^2^), the mean BMI values decreased from 22.26 kg/m^2^ (SD 1.72) at baseline to 21.82 (SD 1.70) at 3 months and 21.74 kg/m^2^ (SD 1.70) at 6 months of HD (P = 0.046). Table 3 presents these results.

**Table 3:** Changes in the levels of TC, LDL-C, HDL-C, non-HDL, TG in subgroups of patients divided according to clinical parameters

	TC mg/dL mean (SD)		Non-HDL mg/dL mean (SD)	LDL-C mg/dL mean (SD)	TG mg/dL mean (SD)
Eutrophy					
Baseline	195.68 (39.14)		142.41 (33.91)	122.47 (31.72)	99.67 (40.69)
3 months	181.82 (40.33)		133.56 (40.98)	108.87 (33.73)	123.40 (105.33)
6 months	177.68 (37.72)		128.08 (39.32)	105.67 (35.16)	112.13(94.00)
P*	0.02		0.09	0.04	0.31
Overweight					
Baseline	198.03 (25.52)		157.70 (27.57)	121.08 (21.59)	178.17 (129.94)
3 months	197.15 (47.14)		150.73 (39.15)	125.00 (35.98)	128.58 (59.10)
6 months	191.71 (48.26)		147.21 (44.51)	117.50 (32.69)	148.33 (112.19)
P*	0.81		0.51	0.63	0.15
Obesity					
Baseline	184.92 (38.13)		136.81 (35.13)	105.12 (33.60)	158.53 (54.39)
3 months	174.91 (40.53)		128.74 (34.32)	98.24 (31.34)	152.53 (75.98)
6 months	173.21 (45.52)		127.50 (39.82)	100.12 (36.15)	136.76 (64.06)
P*	0.31		0.42	0.55	0.28
No hypertension and no diabetes					
Baseline	188.66 (34.32)		140.74 (33.77)	114.74 (32.16)	129.96 (69.89)
3 months	176.31 (31.62)		130.60 (30.50)	104.89 (27.85)	128.56 (86.29)
6 months	172.98 (37.37)		126.91 (37.27)	101.89 (31.75)	125.11 (99.88)
P*	0.01		0.02	0.05	0.92
Hypertension or diabetes					
Baseline	197.72 (36.97)		150.25(32.37)	116.41 (28.82)	165.82 (100.90)
3 months	194.49 (54.53)		145.55 (47.62)	115.94 (42.93)	148.00 (77.55)
6 months	190.57(51.03)		142.87 (45.64)	114.47 (38.95)	141.71 (68.48)
P*	0.73		0.65	0.96	0.37

Eutrophy was defined as BMI < 24.9 kg/m^2^, overweight as BMI > 24.9–29.9 kg/m^2^, and obesity as BMI > 30 kg/m^2^

*BMI* Body mass index, *HDL-C* high-density lipoprotein cholesterol, *LDL-C* low-density lipoprotein cholesterol, *Non-HDL-C* non-HDL cholesterol, *TC* total cholesterol, *TG* triglycerides, *SD* standard deviation

P* values were calculated using ANOVA test

Finally, TC levels and non-HDLC levels significantly decreased from baseline to 6 months with HD therapy regardless of the use of statins at baseline. TC and non-HDL-C levels at baseline and at 3 and 6 months of HD in the subgroups of: patients who used statins (n = 10); and free of statins (n = 34) are shown in Table 4.

**Table 4:** Non-HDL-C and total cholesterol levels at baseline and at 3 and 6 months with HD grouped according to statins use

Statins use	Lipids	Baseline		3 months		**6** months	
		Mean	SD	Mean	SD	Mean	SD
No (n = 34)	Non-HDL-C (mg/dL)	145.14	(34.70)	139.01	(33.89)	132.42	(37.82)
Yes (n = 10)	Non-HDL-C (mg/dL)	141.96	(28.96)	127.44	(51.48)	135.28	(52.53)
No (n = 34)	TC (mg/dL)	192.84	(35.00)	186.77	(38.19)	179.60	(40.07)
Yes (n = 10)	TC (mg/dL)	189.86	(37.80)	171.64	(54.91)	180.38	(55.99)

*Non-HDL-C* non-HDL cholesterol, *TC* total cholesterol, *SD* standard deviation

## Discussion

Treatment of disorders associated with increased risk of cardiovascular events, such as dyslipidemia, is warranted to reduce the risk of thrombosis in APS. In the present study, we observed a high prevalence of cardiovascular risk factors in PAPS patients. The prevalence of smoking or alcohol intake was 15.9%, diabetes was 4.5% and hypertension was 38.6%. Previous studies have also reported a high prevalence of cardiovascular risk factors in patients with APS, as an example, a recent cross-sectional study evaluating 39 patients with PAPS, also found a higher prevalence of hypertension (46.2%), diabetes (12.8%), and smoking (15.4%) [24].

Therapeutic strategies complementary to statin use may be necessary for the management of dyslipidemia in t-PAPS patients, as statins may increase the risk of bleeding in patients taking anticoagulants [12]. A potential complementary therapy for dyslipidemia in PAPS is nutritional intervention with HD. However, nutritional intervention was shown to be less effective in patients with autoimmune diseases. In autoimmune diseases, dyslipidemia may be aggravated by the inflammatory condition of patients, which, in turn, can compromise the effectiveness of diets with lipid restriction [15, 16]. Patients with SLE commonly have higher levels of TG and very low-density lipoprotein (VLDL), associated with lower levels of HDL-C. These lipid disorders are related to the inflammatory activity of the disease, which suggests that SLE leads to an atherogenic lipoprotein profile [25]. A study with 70 SLE patients and controls showed that a nutritional intervention with cholesterol-lowering diet for 12 weeks, was capable of reducing cholesterol and saturated fat intake without, however, reducing lipid levels substantially. In this study, the relative reductions were − 6% for TC, − 2% for LDL-C, − 4% for HDL-C, − 34% for VLDL, and − 24% for TG. The authors concluded that the cholesterol-lowering diet was well accepted and effective in changing patients´ diet and quality of life; however, the effects on serum lipids and lipoproteins were moderate [26].

In our study, HD intake for 6 months was associated with a decrease in the levels of TC (− 7%), non-HDL-C (− 7.85), TG (− 8.54%) and LDL- C (− 7.47%). BMI values were also reduced after HD was initiated. In addition to the reduction in cholesterol levels and BMI, a reduction in carbohydrate, lipid, saturated fat, dietary cholesterol and calories intake combined with an increase in dietary fiber intake was observed. The benefits of HD were more pronounced in the subgroups of patients with increased levels of TC, non-HDL-C, LDL-C or TG at baseline. As an example, after 6 months of HD TC levels were reduced by 7% in patients with normal TC levels at baseline and by 26% in those with high TG at baseline. This may be explained by the fact that HD is not capable of decreasing lipid levels to extremely low levels, as opposed to intensive and moderate intensity statins. Additionally, HD was more effective in patients with obesity, hypertension or diabetes than in those without these comorbidities. Altogether, these results suggest that HD diet can be beneficial for the treatment of PAPS-associated dyslipidemia, particularly in patients without further cardiovascular risk factors.

We also observed that the decrease in lipid levels was accompanied by changes in the diet composition towards a decrease in saturated fat and dietary cholesterol intake and an increase in fiber intake. These observations demonstrate that patients had a good adherence to HD which may have contributed to the decrease in serum lipid levels. Poor diet quality and food habits are among the leading causes of cardiovascular morbidity worldwide [27, 28]. High dietary cholesterol, lipids, and saturated fatty acid intake and low fiber intake contribute to dyslipidemia, obesity, diabetes, and hypertension [27]. Low consumption of polyunsaturated fats has also been associated with increased mortality [29, 30]. Therefore, consumption of saturated fats should be discouraged and partially substituted with polyunsaturated fats, which are associated with decreased levels of TC and LDL-C, as well as decreased cardiovascular events and deaths [29, 30].

Increased dietary cholesterol intake has also been associated with increased levels of serum cholesterol. A clinical study with 56 adult males evaluated the serum cholesterol changes after 6 weeks of controlled lipid intake (0, 106, 212 or 317 mg cholesterol/1000 kcal). The study observed that increased lipid intake resulted in elevated serum cholesterol. The authors described that an increase in cholesterol intake of 100 mg per 1000 kcal of diet resulted in a serum cholesterol level increase by 12 mg/dL. They concluded that dietary cholesterol plays a major role in serum cholesterol levels [31]. Additionally, the National Cholesterol Education Program estimates that diet can lead to 10–15% reduction in blood cholesterol levels. There is now a consensus that diets should be low in total lipids, cholesterol, and saturated fatty acids in order to maintain low levels of blood cholesterol [23, 32, 33].

It is also worth noting that dietary fiber intake increased after HD was initiated. Several studies have demonstrated that dietary fiber intake is associated with reduced levels of cholesterol. In a clinical study, Chandalia et al. [34] demonstrated that high fiber intake reduced serum lipid concentrations, improved glycemic control, and reduced hyperinsulinemia in type 2 diabetic patients. The findings of this study provided evidence for the recommendations of soluble and insoluble fiber-rich foods intake, such as whole grains, fruits, and vegetables, for the primary prevention of cardiovascular diseases [34].

Some limitations of our study need to be discussed. First, this was a single arm study and we cannot exclude the possibility of a placebo effect or changes in lifestyle having affected the results. However, patients did not report changes in lifestyle or physical activity during the study. Second, the sample size was small, which can be explained by the relative rarity of thrombotic APS. However, the number of patients included was sufficiently powered to demonstrate the effect of HD on lipid levels in this population. Third, the study follow-up time was short and a longer follow-up could have possibly led to a better nutritional adequacy of the diet and changes in lifestyle. Fourth, adherence to HD was evaluated using a 24-h dietary recall. This approach is reasonable to evaluate nutritional adequacy of the diet, however the approach may not be accurate as the evaluation relies on patients' self-reported information. Fifty, despite the decrease in dietary lipid and fiber intake with HD, diet adequacy was not optimal given that the carbohydrate intake also increased during the study. Sixty, this study is underpowered to evaluate thrombotic outcomes due to the short-term follow-up, therefore clinical studies aimed at determining the impact of lipid lowering strategies on the risk of thrombosis in APS are needed. Finally, although the results from subgroup analyses suggested that HD may have the strongest potential to decrease lipid levels in patients with no other comorbidity besides t-PAPS and dyslipidemia, these analyses must be interpreted with caution as the study was not designed to analyze differences in subgroups.

In conclusion, HD was well tolerated by t-PAPS and led to diet adequacy, with lower dietary lipids and higher fiber intake. Diet adequacy was accompanied by a decrease in the levels of plasma lipids and in BMI values. These findings demonstrate that nutritional intervention is feasible among PAPS patients with thrombosis and may contribute to the treatment of dyslipidemia, a condition that worsens these patients' prognosis. Even though treating dyslipidemia may improve APS prognosis, further studies are necessary to evaluate whether lipid lowering strategies play a role in preventing thrombotic events in APS.
